# Abnormal White Matter Integrity in the Corpus Callosum among Smokers: Tract-Based Spatial Statistics

**DOI:** 10.1371/journal.pone.0087890

**Published:** 2014-02-07

**Authors:** Wakako Umene-Nakano, Reiji Yoshimura, Shingo Kakeda, Keita Watanabe, Kenji Hayashi, Joji Nishimura, Hidehiko Takahashi, Junji Moriya, Satoru Ide, Issei Ueda, Hikaru Hori, Atsuko Ikenouchi-Sugita, Asuka Katsuki, Kiyokazu Atake, Osamu Abe, Yukunori Korogi, Jun Nakamura

**Affiliations:** 1 Department of Psychiatry, University of Occupational and Environmental Health, Kitakyushu, Japan; 2 Department of Radiology, University of Occupational and Environmental Health, Kitakyushu, Japan; 3 Department of Psychiatry, Kyoto University Graduate School of Medicine, Kyoto, Japan; 4 Department of Radiology, School of Medicine, Nihon University, Tokyo, Japan; “Mario Negri” Institute for Pharmacological Research, Italy

## Abstract

In the present study, we aimed to investigate the difference in white matter between smokers and nonsmokers. In addition, we examined relationships between white matter integrity and nicotine dependence parameters in smoking subjects. Nineteen male smokers were enrolled in this study. Eighteen age-matched non-smokers with no current or past psychiatric history were included as controls. Diffusion tensor imaging scans were performed, and the analysis was conducted using a tract-based special statistics approach. Compared with nonsmokers, smokers exhibited a significant decrease in fractional anisotropy (FA) throughout the whole corpus callosum. There were no significant differences in radial diffusivity or axial diffusivity between the two groups. There was a significant negative correlation between FA in the whole corpus callosum and the amount of tobacco use (cigarettes/day; R = − 0.580, p = 0.023). These results suggest that the corpus callosum may be one of the key areas influenced by chronic smoking.

## Introduction

The most prevalent substance dependence issue worldwide is tobacco smoking. Smoking leads to serious public health problems and preventable early deaths [1]. In neuroimaging researches, smoking has been associated with large-scale structural brain alterations [2], [3], [4]. Particularly, recent voxel-based morphometry (VBM) study, smoking was associated with reductions in cerebral grey matter in the prefrontal, anterior cingulate, parietal, and temporal cortices and the cerebellum [3] [4]. There was an inverse relationship between cortical volume or cortical thickness and exposure to smoking [3] [5]. Furthermore, lower gray matter density in the prefrontal cortex and higher density in the insula have also been observed in smokers [6]. In short, the results of previous studied were consistent.

Diffusion tensor imaging (DTI) is sensitive to water diffusion characteristics and has been developed as a tool for investigating the local properties of brain white matter [7]. There are three diffusion metrics derived from DTI data: fractional anisotropy (FA), which reflects the directionality of water diffusion and the coherence of white matter fiber tracts; axial diffusivity (AD), which measures the magnitude of diffusivity along the principle diffusion direction; and radial diffusivity (RD), which reflects the magnitude of diffusivity perpendicular to the principle diffusion direction [8] [9]. Furthermore, the mean diffusivity (MD) was also calculated from DTI data. In a previous study, higher FA was measured in the corpus callosum of smokers compared with age-matched nonsmokers [10]. Jacobsen et al. [11] examined FA among adolescent smokers and nonsmokers with and without prenatal exposure to maternal smoking. In this study, both prenatal exposure and adolescent exposure to tobacco smoke was associated with increased FA in the anterior cortical white matter. Adolescent smoking was also associated with increased FA in regions of the internal capsule that contain auditory thalamocortical and corticofugal fibers. Thus, the effect of smoking on FA was inconsistent, and the regions affected were various.

The novel voxel-wise approach of tract-based spatial statistics (TBSS) was recently introduced [12], restricting the evaluation of diffusion parameters to a white matter skeleton common to all subjects. Voxel-based DTI analysis is not a mainstream of SPM (Statistical Parametric Mapping; Institute of Neurology, London, UK) and not officially supported. Therefore, there has not been a consensus about the method to spatially normalize FA images and the size of the smoothing kernel. To overcome these problems TBSS has been proposed [12].

To date, only two studies existed investigated white matter in smokers using TBSS. First, Zhang et al. [13] investigated FA using TBSS analysis methods in smokers with schizophrenia and healthy age-matched control subjects. The authors reported lower FA in the prefrontal white matter of a subsample of highly nicotine-dependent smokers and a negative correlation between FA and nicotine dependence, as measured by the Fagerström score. Second, Lin et al. [14] also reported that compared with nonsmokers, heavy smokers had lower FA in the left anterior corpus callosum. The results of two former studies were controversial, and the influence of smoking on white matter still remains unclear.

The objectives of the current study were 1) to investigate differences between smokers and nonsmokers in white matter, and 2) to examine relationships between white matter integrity and nicotine dependence parameters in the smokers.

## Methods

### Participants

Twenty smokers met the DSM-IV criteria for nicotine dependence; the score on the Tobacco Dependence Screener Scale (TDS) was used to diagnose nicotine dependence [15] The exclusion criteria included a current or past history of any comorbid neurological disorder, significant medical conditions, abnormal results on laboratory screening tests, or addiction to alcohol or other substances (with the exception of nicotine). We also performed the Mini International Neuropsychiatric Interview (MINI) [16] to rule out past or present history of comorbid psychiatric disorders. Finally, nineteen males were enrolled of this study. As part of the initial clinical evaluation, all smokers were asked to complete baseline questionnaires that assessed detailed demographic information and smoking-related clinical variables. The expiratory carbon monoxide (CO) levels of the smokers were measured using the Smokerlyzer system (Bedfont Scientific Ltd., Rochester, UK). Nicotine dependence levels were assessed with the Fagerström Test for Nicotine Dependence (FTND) [17]. Twenty age-matched nonsmokers with no current or past psychiatric history were included as controls. Finally, eighteen males were enrolled of this study. Alcohol use levels were assessed with the Alcohol Use Disorders Identification Test (AUDIT) [18] [19]. This study was approved by the Ethics Committee of the University of Occupational and Environmental Health. All the participants gave written informed consent to participate in the study.

### Diffusion Tensor Images: Magnetic Resonance Imaging Scanning Protocol

All MR examinations were performed with a 3T MR system (Signa EXCITE 3T; GE Healthcare, Milwaukee, WI) with an 8-channel brain-phased array coil. The brain volume data were obtained with a three-dimensional fast spoiled gradient recalled acquisition with steady state (3D-FSPGR), which was acquired with parameters of 10/4.1/700 (repetition time msec/echo time msec/inversion time msec), a flip angle of 10 degree, a 24-cm field of view, and 1.2-mm-thick sections with 0.47×0.47×1.2 mm resolution. Diffusion tensor images were acquired using a single-shot, spin-echo echo-planar sequence with the following parameters: TR/TE = 12000/83.3 msec, 4-mm slice thickness, no gap, 26-cm field of view; number of excitation = 1, and a spatial resolution of 1.02×1.02×4 mm. Diffusion gradients (b value of 1,000 sec/mm^2^) were always applied on each axis simultaneously around the 180-degree pulse. The diffusion properties were measured along 25 noncollinear directions. The structural distortion of diffusion-weighted MR images was corrected based on each T2-weighted planar image (b = 0 sec/mm^2^) [20].

### Volume Imaging Processing

All images were corrected for image distortion caused by gradient nonlinearity using “GradWarp” [21] and for intensity inhomogeneity using “N3” [22]. Image processing for VBM, a fully automatic technique for the computational analysis of differences in regional brain volume throughout the entire brain, was conducted using SPM8. The 3D-FSPGR images in native space were spatially normalized, segmented into grey matter (GM), white matter (WM), and cerebrospinal fluid images, and intensity-modulated using the Diffeomorphic Anatomical Registration Through Exponential Lie Algebra (DARTEL) toolbox on SPM8 [23]. Age was treated as confounding covariates. The DARTEL was proposed by Ashburner as an alternative method of normalization in the SPM package [24]. In an intensity modulation step, the voxel values of the segmented images were multiplied by the measure of warped and unwarped structures derived from the nonlinear step of the spatial normalization. This step converted the relative regional GM and WM density into absolute volume, expressed as the amount of per-unit volume of brain tissue before spatial normalization. The resulting modulated GM and WM images were smoothed with an 8-mm Gaussian kernel. An analysis of covariance model was used to examine group differences in GM and WM, with age as a covariate. These analyses yielded statistical parametric maps {SPM (t)} based on a voxel-level height threshold of *p*<0.001. Topological false discovery rate (FDR) correction was applied. The significance level was set at FDR-corrected *p*<0.05.

### DTI Image Processing using TBSS

FA maps were computed for all subjects from the diffusion-tensor images after eddy current correction and automatic brain extraction using the FMRIB Diffusion Toolbox, which is part of the Functional MR Imaging of the Brain Software Library (FMRIB) Software Library [25]. The data were corrected for spatial distortion caused by gradient nonlinearity using grad_unwarp [21]. A voxel-wise statistical analysis of the DTI data was performed using TBSS [12] and implemented in the FMRIB Software Library 4.1.6 (University of Oxford, Oxford, UK) [25]. The FA, AD, RD and MD images were created by fitting a tensor model to the raw diffusion data. Brain extraction was performed using the Brain Extraction Tool 2.1 [26]. The FA data for all the subjects were aligned into a common space via nonlinear registration [27]. Next, a mean FA image was created and thinned to create a mean FA skeleton representing the centers of all tracts common to the group. This skeleton had a threshold of FA>0.20. Each subject’s aligned FA data were then projected onto this skeleton, and the resulting data were fed into a voxel-wise cross-subject statistical analysis. Subsequently, other relevant DTI output images (AD, RD, MD) were projected onto the mean FA skeleton to compare other diffusivity values between groups in the same spatial location.

Diffusion tensor metrics were compared between groups with unpaired *t* test adjusted for the subject’s age using TBSS analysis. The significance threshold for between-group differences was set at *p*<0.05; this was corrected for multiple comparisons across voxels using the threshold-free cluster-enhancement option. The number of permutations was set to 20,000 in all voxel-wise analyses.

### DTI Imaging Process Tract-Specific Analysis

FA maps were computed using dTV II and VOLUMEONE1.72, developed by Masutani et al. [28] (University of Tokyo; diffusion tensor visualizer available at http://www.volume-one.org/). Diffusion tensors were computed, and fiber tracts were created by interpolation along the z-axis to obtain data (voxel size 2.0×2.0×3.0 mm^3^). Color coded maps were created using 26 sets of images (25 sets with b = 1,000 s/mm^2^, 0 set with b = 0 s/mm^2^). Fiber tracts were based on a fiber assignment that was made using the continuous tracking approach [29] to obtain a three-dimensional tract reconstruction. The fiber tracts were initiated by placing a seed area in the anatomical regions through which the particular fibers were expected to course [26]. Tract measurements of the corpus callosum were performed by one of the authors (K.W.), who was blind to the status of each subject. The seed ROI was placed manually, including the entirety of the corpus callosum, on a reconstructed midsagittal image with a non-diffusion-weighted image (b = 0 s/mm^2^). Tractographic images of the corpus callosum were generated with threshold values of line-tracking termination FA>0.18. Voxelization along the corpus callosum was then performed. To reduce the partial volume effect of the peripheral portion of the tract and to eliminate small incidental artifactual lines, we used a shape-processing technique based on mathematical morphology [28]. In this shape-processing technique, we dilated the voxels once and eroded them twice. The FA values in coregistered voxels were calculated.

### Statistical Analysis

All statistical analyses were conducted using SAS Version 9.3 (SAS Institute, Cary, North Carolina, USA). The Mann-Whitney U test and the chi-squared test were used to compare the variables between the smokers and nonsmokers. Pearson’s correlation coefficient was used to examine the relationship between FA levels and smoking-related clinical variables. The level of significance was set at *p<*0.05.

## Results

### Subject Demographics

Table 1 shows the backgrounds of the smokers (N = 19) and the nonsmokers (N = 18). All were males and there was no significant difference in the distributions of age (Smokers: 40.5±8.6, Nonsmokers: 40.5±8.6) Detailed information about smoking-related clinical variables and the AUDIT scores was not available for three subjects (N = 16). The mean AUDIT score for the smokers was significantly higher compared to that of the nonsmokers (smokers: 9.1±6.3, nonsmokers: 4.9±4.4; *p* = 0.022).

**Table 1:** Background of smokers and non-smokers.

	Smokers (N = 19)*	Non-Smokers (N = 18)	P
Age (years)	40.5±8.6 (27–54)	36.4±8.0 (30–61)	0.094
AUDIT	9.1±6.3	4.9±4.4	0.022
Age of first use (years)	19.6±1.4		
Duration of use (years)	19.8±8.5		
Amount of use (cigarettes/day)	18.8±7.4		
Brinkman index (daily number of cigarettes × year)	394.1±271.0		
Scores of TDS	6.9±1.7		
Scores of FTND	3.7±1.5		
CO levels (ppm)	16.4±12.8		
Total gray matter volume	718.5±52.9	738.5±52.1	0.186
Total white matter volume	541.2±46.8	545.2±39.5	0.750

Abbreviation. AUDIT: Alcohol Use Disorders Identification Test; TDS: Tobacco Dependence Screener Scale; FTND: Fagerström Test for Nicotine Dependence; CO; carbon monoxide.

*Detailed information about the smoking-related clinical variables and the AUDIT scores of three subjects (N = 16) were not available. The mean AUDIT scores of the smokers (N = 16) was significantly higher than that of the nonsmokers (N = 18; smokers: 9.1±6.3; nonsmokers: 4.9±4.4; p = 0.022).

### Total Volumes

There was no significant difference in the global brain volume (total gray matter and total white matter) between the smokers and the nonsmokers (Table 1). The regional volumetric analysis showed no significant group difference in any regions.

### TBSS and Tract-Specific Analysis Results

**Figure 1:**
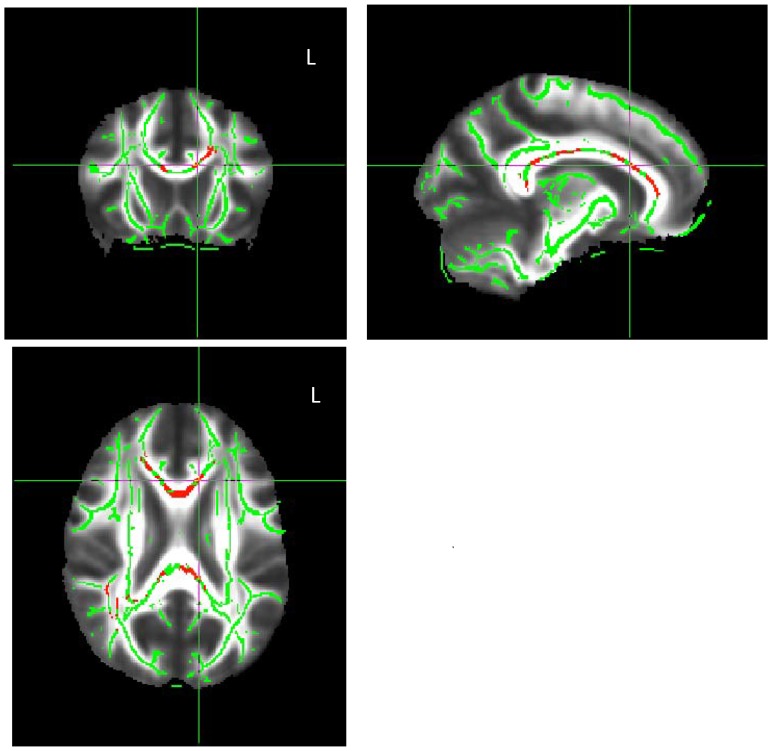
TBSS and tract-specific analysis results. The red voxels represent the areas where the fractional anisotropy (FA) levels of smokers were significantly reduced with respect to those of nonsmokers. Compared with nonsmokers, the smokers exhibited a significant decrease in FA throughout the whole corpus callosum.

**Figure 2:**
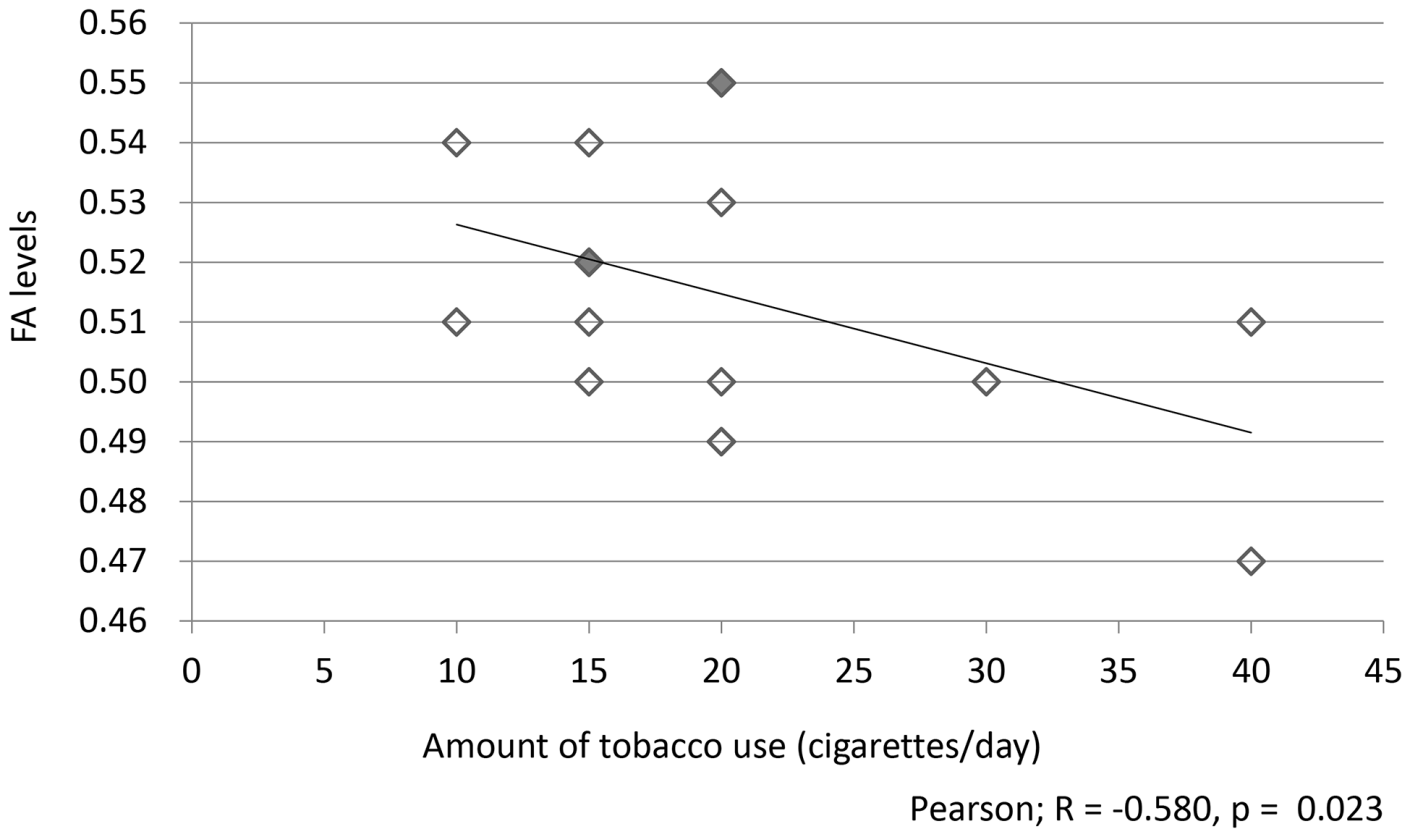
The correlation between FA levels throughout the whole corpus callosum and the amount of tobacco use (N = 16). There was a significant negative correlation between FA levels throughout the whole corpus callosum and the amount of tobacco use covariant age (cigarettes/day; R = − 0.580, *p* = 0.023). The number of spots was decreased from 16 to 13 because three spots represented the same data (three represented the FA level = 0.52 and N = 15, and two represented the FA level = 0.55 and N = 20; these spots are shown in gray spots).

Compared with the nonsmokers, the smokers exhibited a significant decrease in FA throughout the corpus callosum (Figure 1). There were no significant differences in AD or RD. In addition, there was a significant negative correlation between FA throughout the whole corpus callosum and the amount of tobacco use (cigarettes/day) covariant age (R = −0.580, *p* = 0.023; Figure 2), but there were no significant correlations between the amount of tobacco use and AD, RD or MD (*p* = 0.522, *p* = 0.678, *p* = 0.850). No significant correlations were observed between FA and the duration of tobacco use, the Brinkman index, the TDS scores, the FTND scores, CO levels, or the mean AUDIT scores.

## Discussion

The current study provides novel evidence of decreased FA throughout the whole corpus callosum in smokers. Based on this result, we evaluated the relationship between FA levels of corpus callosum and smoking-related clinical variables using tract-specific analysis. This analysis has the advantage of detecting abnormalities in specific white matter tracts, whereas TBSS can influence the outcome by multiple comparisons [30]. We found a significant inverse correlation between FA levels and the amount of tobacco consumptions. No significant differences regarding AD, RD or MD in any region of brain were observed. Our finding is concordant with the results of Lin et al. [14], which observed decreased FA in the left anterior corpus callosum, but not concordant with a significantly decreased AD and a significantly increased RD. It is generally believed that AD mainly reflects axonal integrity [31] and RD is more related to the integrity and thickness of the myelin sheaths covering the axons [32]. Animal DTI studies have demonstrated that decreased AD with the degeneration, and increased RD with demyelination [32], [33]. And the decreased FA in corpus callosum of smokers is likely the manifestation of axonal damage and disrupted myelin integrity in the region. Our subjects were lower FTND score rather than Lin et al. [14], which suggests our samples were not heavy smokers. In other words, not much amount of smoking might influence only in FA.

Zhang et al. [13] also reported lower-than-normal FA in a section of the left prefrontal white matter in a subsample of highly dependent smokers, and no smoking-related differences in other white matter areas. This inconsistency may arise from the comorbidity of psychiatry disorder in a part of our samples both smokers and healthy controls.

The corpus callosum connects the brain hemispheres and facilitates communication between left- and right-side brain structures; it is the largest white matter fiber tract connecting the neocortex of the two hemispheres [34]. The anterior parts of the corpus callosum connect the frontal cortices, while the body and the splenium connect the parietal, temporal, and occipital homotopic regions [35]. Compromised fiber connectivity within the corpus callosum has been previously reported in subjects with substance addictions [36]. Decreased FA in the genu, body and splenium of the corpus callosum has been associated with alcohol-dependent subjects [37]. Significant reductions in FA in the genu and rostral body [38] and in the body and splenium of the corpus callosum [39] have been reported in cocaine-dependent subjects. Methamphetamine abusers showed reduced white matter integrity in the genu [40] and the rostral body [41] of the corpus callosum. There was a significant reduction in FA in the genu and the isthmus of the corpus callosum in opiate-dependent subjects [42]. Qiu et al. [43] recently reported that heroin-dependent individuals had significantly lower FA throughout the brain, including the genu and the isthmus of the corpus callosum, compared with controls. Taken together, these findings suggest that substance abuse may be associated with reduced FA levels that reflect less microstructural integrity. In contrast, Paul et al. [10] reported that smokers exhibited higher levels of FA in the corpus callosum than nonsmokers did. The authors hypothesized that either nicotine-induced cytotoxic cell swelling secondary to nicotine-induced osmotic imbalances or vasogenic swelling characterized by plasma fluid leaking into the parenchymal interstitial space might lead to alterations in the white matter. According to Hudkins et al. [44], FA may rise during the early years of smoking and subsequently decline with continued smoking in later years. Negative correlations between the age at which the subjects began smoking and FA have been observed [41]. Our finding in the present study is in accordance with that of Hudkins et al. [41]. In short, it is plausible that nicotine bidirectionally influences FA in the corpus callosum based on smoking duration or/and smoking amount. The subjects of Lin et al. [14] were also older ages. The reason of discrepancy about location of corpus callosum between whole corpus callosum in this study and anterior corpus callosum in Lin et al. [14] study might be differences sample characteristics (our subject was only male), level of cigarette smoking (our subject was lower FTND score) and alcohol consumption.

There were several limitations to our study. First, the sample size was small and this might lead absence of finding a change in the frontal cortex in the RD and AD Second, we did not evaluate healthy controls using any structured interviewed. Therefore, we might not completely exclude comorbidity psychiatric disorders among both samples. Third, the results may have been influenced by alcohol intake, given the higher AUDIT scores among the smokers (Table 1) and we could not evaluate comparing two groups covariant with scores of AUDIT. The level of score of AUDIT among smokers did not correspond to alcoholism, and there was not a significant correlation between FA level and mean AUDIT scores. Taking together, it is not ruled out that cigarette smoking increases alcohol consumptions, which might influence the results. Forth, we did not measure plasma levels of nicotine and cotinine, a metabolite of nicotine. We are currently undergoing the new study taking account of above problems.

In conclusion, we found that FA in smokers was significantly decreased compared with that of nonsmokers. Moreover, an inverse correlation between the amount of cigarette smoking and FA throughout the whole corpus callosum, which may shed light on the vulnerability of the region after long-term exposure in nicotine.
